# Animal models and systems biology approaches for the functional validation of genetic determinants of skeletal diseases

**DOI:** 10.1186/1471-2474-16-S1-S18

**Published:** 2015-12-01

**Authors:** Peter A Bell, Michael D Briggs

**Affiliations:** 1 Institute of Genetic Medicine, Newcastle University, Newcastle, UK

## 

Rare skeletal diseases are a diverse group of diseases that primarily affect development of the skeleton. There are more than 450 unique phenotypes that, although individually rare, have an overall prevalence of at least 1 per 4,000 children. Pseudoachondroplasia (PSACH) and multiple epiphyseal dysplasia (MED) are skeletal diseases caused by missense mutations/deletions in the genes encoding important cartilage extracellular matrix proteins (ECM), and are characterized by disproportionate short stature, joint pain and early-onset osteoarthritis.

In-depth characterization of MED and PSACH mouse models has revealed that endoplasmic reticulum (ER) stress, reduced cell proliferation and abnormal ECM assembly are important pathological consequences of mutant protein expression. Ongoing work aims to consolidate data from other models of skeletal disorders using a systems biology approach as part of the EU FP7 SYBIL (Systems biology for the functional validation of genetic determinants of skeletal diseases) project, in order to gain a mechanistic understanding of disease processes and to deliver new and validated therapeutic targets.

Key to delivery of new targets and therapies is the identification of relevant disease biomarkers, which will allow the monitoring of responses to therapeutic interventions. This is particularly critical for skeletal diseases, for which biopsy material is not readily accessible. We have identified differences in the extractability of a number of ECM components from the cartilage of MED and PSACH mouse models, relative to controls. The differences in extractability of these proteins (which include FACIT collagens (types XII and XIV), tenascins (C and X), and fetuin A) may represent differences in the stability of these proteins within the cartilage ECM, which might potentially be exploited for use as biomarkers of disease progression. We are currently using biochemical and mass spectrometry analysis of easily obtained biological samples such as blood, urine and cell culture medium in order to identify and validate novel biomarkers for skeletal diseases.

